# Hepatic Hemangioma in Celiac Patients: Data from a Large Consecutive Series

**DOI:** 10.1155/2015/749235

**Published:** 2015-01-12

**Authors:** Sara Massironi, Federica Branchi, Roberta Elisa Rossi, Mirella Fraquelli, Luca Elli, Maria Teresa Bardella, Federica Cavalcoli, Dario Conte

**Affiliations:** ^1^Gastroenterology and Endoscopy Unit, Fondazione IRCCS Cà Granda Ospedale Maggiore Policlinico, Via Francesco Sforza 28, 20122 Milan, Italy; ^2^Department of Pathophysiology and Transplantation, Università degli Studi di Milano, Via Francesco Sforza 35, 20122 Milan, Italy

## Abstract

*Background and Aims*. Hepatic hemangioma (HH) has a widely ranging prevalence. The etiology is unclear; however, associations with autoimmune disorders have been described. We aimed at evaluating the prevalence of HH in celiac disease. *Methods*. Ninety-seven consecutive patients with celiac disease (18 M, 79 F, median age 41, and range 17–84 years) underwent liver ultrasound between January 2011 and 2012. The findings were compared with those of 1352 nonceliac patients (581 M, 771 F, median age 50, and range 16–94 years), without liver disease or previously detected HH, who underwent US in the same period. *Results*. Ultrasonographic findings consistent with HH were observed in 14 celiac patients (14.4%), a prevalence significantly higher than in controls (69 cases, 5.1%) (*P* = 0.0006). Subgroup analysis showed that, among women, the prevalence of HH was 16.4% in the celiac disease group (13/79) compared with 5.9% in controls (46/771) (*P* = 0.002). In celiac setting, HH had a median diameter of 1.3 cm and presented as a single lesion in 12 cases (86%). *Conclusions*. Our findings are consistent with a significantly higher prevalence of HH in celiac patients. Although mechanisms underlying this association remain unclear, autoimmune and metabolic processes, as well as alterations of gut-liver axis equilibrium, could play a role.

## 1. Introduction

Hepatic hemangioma (HH) is the most common benign mesenchymal hepatic tumor. It has a prevalence ranging from 3 to 20%. The prevalence is highest in women [1, 2], and in most cases it is an incidental or autopsy finding [2, 3]. The pathogenesis is far from being clearly understood, although they are supposed to develop as a consequence of unregulated angiogenesis [4], influenced by either estrogen levels [5, 6] or genetic predisposition [7–12]. An association of HH with systemic lupus erythematosus (SLE) has also been reported, suggesting that HH might be considered a hepatic manifestation of autoimmune diseases [4].

HHs have never been reported in association with celiac disease (CD), an immune-mediated enteropathy caused by permanent immunological intolerance to ingested gluten. Celiac disease occurs in genetically predisposed people and affects approximately 1% of the population in Western countries [13–15]. Many pathological conditions such as malignancy have been reported as a possible association with CD [16–22]. Hepatic disorders are common in CD [23, 24] and include steatosis, cryptogenic hypertransaminasemia [25], and autoimmune liver diseases [26]. Malabsorption [27], increased intestinal permeability [28, 29], bacterial overgrowth [30], and intestinal inflammation [31] have been reported as of relevance in the pathogenesis of liver injury in CD.

Alteration of the gut-liver axis equilibrium plays a major role in the development of immune disorders involving the small bowel and liver [32, 33]. In celiac disease, the increased intestinal permeability may cause an increased flow of molecules to the liver through the portal circulation. These molecules can originate from the local immune reaction and the cross-linking between tissue transglutaminase and food or bacterial antigens [34]. There is high probability that chronic liver exposition to active molecules could lead to the development of certain liver diseases. Exposure of the liver to immunologically active molecules generated in the small bowel mucosa of CD patients or to high levels of angiogenic factors, such as vascular endothelial growth factor (VEGF), could, for instance, lead to the development of vascular lesions such as HH. An overexpression of mucosal VEGF in CD patients, as already suggested in SLE [4], would be an attractive hypothesis.

As a consequence of previous observations made during routine clinical practice and based on data collected during a previous study by our group [35], we developed a hypothesis that HH could be a frequent finding in patients with CD. We have performed a retrospective analysis of a consecutive series of patients, with the aim of evaluating if the prevalence of HH in a celiac population was higher than that expected in the general population. This could lead to the underlining of a possible association with a multifactorial basis between HH and CD.

## 2. Methods

The study was carried out at the Gastroenterology and Endoscopy Unit, Fondazione IRCCS Ca' Granda, Ospedale Maggiore Policlinico, Milan (Italy).

A series of 97 consecutive patients affected by CD who were prospectively enrolled for another study by our group between January 2011 and January 2012 [35] underwent a complete abdominal ultrasound (US) as part of a thorough clinical evaluation.

US liver findings were compared with those of a consecutive series of nonceliac patients referred for abdominal US for unspecific gastrointestinal symptoms (i.e., dyspepsia, abdominal pain, and diarrhea) by our gastroenterology outpatient clinic during the same period.

All subjects gave their written informed consent to the study, which was approved by the local ethics committee.

### 2.1. Patients with CD

The diagnosis of CD was made according to currently accepted criteria [36] and was based on both serology and histology. Anti-endomysial antibodies were sought using direct immunofluorescence on monkey esophagus (Bio-Rad, Milan, Italy); positive staining around the smooth muscle was considered positive. Anti-transglutaminase antibodies were assessed by ELISA (Eurospital, Trieste, Italy); titres above 7 arbitrary units were considered positive [37, 38]. During upper gastrointestinal tract endoscopy, four biopsies were taken from the second portion of the duodenum and the histological diagnosis of CD was made according to the Marsh classification modified by Oberhuber et al. [39, 40]. All CD patients underwent complete abdominal US and clinical and laboratory parameters were collected.

### 2.2. Control Subjects

The control group consisted of consecutive nonceliac patients with persistently normal transaminases and no known liver diseases, who underwent abdominal US for unspecific gastrointestinal symptoms (i.e., dyspepsia, abdominal pain, and diarrhea) during the same period. Exclusion criteria for both groups included previously detected HH and/or presence of known liver disease (including chronic hepatitis B virus and hepatitis C virus infection, liver cirrhosis, and nonalcoholic steatohepatitis). For control subjects, additional exclusion criteria were known CD or suspected CD, defined as presence of either serological positivity or pathologic histology at the time of abdominal US.

### 2.3. US Studies

Abdominal US examinations were performed after an overnight fast, with a Philips iU22 system (Philips Ultrasound, Bothell, WA, USA), provided with a multifrequency convex transducer (C5–2, 5–2 MHz). A complete, standard gray-scale US examination of the upper abdomen was warranted for all patients. All examinations were obtained and evaluated in real time by two expert operators (MF and SM).

### 2.4. Diagnosis of HH

HHs usually have typical sonographic features of round-shaped hyperechoic lesions although it may appear as a lesion with irregular shape or with hypoechoic pattern, especially in the context of liver steatosis.

After a first US finding of HH, all patients referred to our clinic underwent US controls after 3 and 6 months in order to confirm the diagnosis and to assess stability of shape and size.

In cases where the sonographic features of the HH findings were not typical, patients underwent further radiological examination with magnetic resonance (MRI).

### 2.5. Statistical Analysis

The results are expressed as median and range, unless otherwise stated. All data were tested for a normal distribution using the Kolmogoroff-Smirnoff test. Differences between percentages were evaluated using Fisher's exact test. Differences between groups were evaluated by means of the Mann-Whitney and Kruskal-Wallis tests, followed by Dunn's multiple comparison test when appropriate. Correlation between variables was assessed by determining Spearman's coefficient. A *P* value < 0.05 was considered statistically significant.

## 3. Results

Ninety-seven consecutive patients (18 M, 79 F, median age 41, and range 17–84 years) with confirmed CD were included in the study. Most of them (72, i.e., 74.2%) were on an ongoing gluten-free diet regimen for a mean period of five years (range 1–31). A total of 1352 consecutive nonceliac patients (581 M, 771 F, median age 50, and range 16–94 years) with persistently normal transaminases and no known liver diseases were included as control group. Demographic and clinical characteristics of the study groups are detailed in Table 1.

In 14 of the 97 celiac patients (14.4%) US findings were fully consistent with HH, compared with 69 (5.1%) in the control group (*P* = 0.0009).

As detailed in Table 2, HHs in celiac patients had a median diameter of 1.3 cm (range 0.6–6.8 cm) and presented as a single lesion in 12 of the 14 patients (86%). They appeared as hyperechoic lesions in 13 patients (93%), without significant differences from the US findings in the control group, where the median diameter was 1.5 cm (range 0.7–4.6) and the lesion was single in 59 cases (86%). In cases where the sonographic features of the HH findings were not typical (i.e., atypical shape, hypoechoic pattern), patients underwent further radiological examination with magnetic resonance (MRI), which confirmed the diagnosis (1 patient in the CD group and 11 patients in the control group).

The prevalence of female gender was higher in CD than in the control population (*P* < 0.0001). The two groups were indeed similar in regard to age and presence of steatosis (the latter was present in 37% of CD patients versus 34.6% of control subjects, *P* = n.s.).

**Figure 1:**
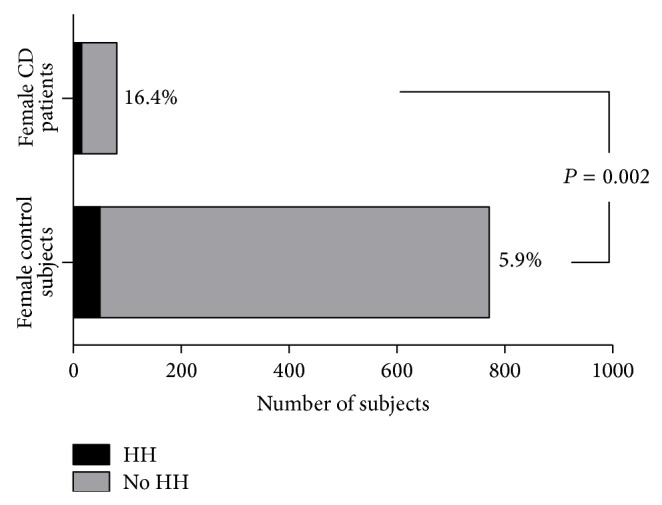
Prevalence of hepatic hemangioma (HH) in female CD patients and in female control subjects. *y*-axis: female control subjects and female CD patients; *x*-axis: number of subjects.

The prevalence of HH was 5.6% in male celiac patients (1/18) compared with 3.9% in nonceliac patients (23/581) (*P* = n.s.). Among women, the prevalence was 16.4% in the CD group (13/79) versus 5.9% in the control group (46/771) (Figure 1, *P* = 0.002).

## 4. Discussion

Results from this series suggest a higher HH prevalence in CD than in controls, delineating a previously unreported association. Indeed, the prevalence of HH at US liver scan was about three times higher in CD than in controls, the difference being even higher in women.

This study represents the first series aimed at evaluating the possible association between HH and CD. In this context, it is of relevance that both the large control group and the inclusion and exclusion criteria make this group highly representative of the general population. Firstly, patients with existing liver disease were excluded from the study because the sonographic diagnosis of HH would have been extremely difficult in this specific setting, requiring more than one imaging technique to avoid a misdiagnosis of liver malignancy. Additionally, patients with already known HH were excluded, due to the risk of bias from extremely high prevalence of patients with known focal liver lesions referred to our tertiary center for consultation and repeat US scans. Excluding these patients, the observed prevalence of 5.1% of US findings of HH in the control group is consistent with epidemiological data in the general population [1–3].

A point of weakness of this series is the relatively small number of male subjects in the CD group. In fact, both groups had a higher prevalence of female subjects, particularly relevant in the CD group (79 versus 18), reflecting the higher prevalence of the disease in females [41, 42]. The finding of only one HH among the 18 male CD patients precludes any conclusions. This suggests the need for collecting a larger number of male CD patients in future series, to both improve the statistical power of US findings and help in clarifying the potential influence of estrogen.

A second aspect of weakness is that at present MRI is generally considered the reference standard for detecting HH. In this study MRI was actually performed only when facing large HHs or lesions with atypical features. There are conflicting recommendations regarding the management of patients with US findings consistent with “typical” HH and it is a matter of discussion whether the diagnostic accuracy of US is sufficient for a correct diagnosis of HH, mainly in the setting of patients with liver steatosis, which was detected in more than one-third of CD patients and controls. Aiming at increasing the diagnostic performance of US liver scan, US examinations were performed by two operators (MF and SM) with large experience in the field.

Another point to consider is the prevalence of single HH at US. In both CD patients and controls HH was detected as a single lesion in nearly 86% of the cases. It is commonly believed that HH is often solitary, but available data support the hypothesis that multiple lesions could be present in up to 40% of patients [1, 2]. However, as scattered data are available about the US detection rate of multiple versus single lesions, a 14% rate of detection of multiple HH in both groups, without a significant difference, suggests a satisfying accuracy for this imaging technique.

The mechanisms underlying the possible association between HH and CD are still far from clearly understood. However, hormonal, autoimmune, genetic and metabolic processes could play a role.

Firstly, HH and CD are known to be more common in females. This could partially explain the association of CD and HH detected in our study, suggesting a potential role of estrogen as triggering factor. However, we also observed an increased prevalence of HH in the CD male group (5.6% versus 3.9%), although the data did not reach statistical significance. Moreover, estrogen receptors have not been found in all HHs, and hemangioma growth may also occur in postmenopausal women, independent of hormonal influence [3].

Secondly, Berzigotti et al. reported fivefold increased odds of HH in a series of SLE patients, suggesting that HH might be considered among the hepatic manifestations of SLE [4]. Since SLE is considered an autoimmune disorder, as is CD, autoimmunity could also play an important role. However, the exact autoimmune mechanisms potentially involved remain to be elucidated.

Moreover, as already suggested in SLE [4], an overexpression of angiogenic factors, particularly vascular endothelial growth factor (VEGF), in CD patients would be an attractive hypothesis even if further studies are needed in this context. It has been suggested that hemangiomas could be related to a dysregulated angiogenesis due to an imbalance between angiogenic and angiostatic factors [43]. The alteration of gut-liver axis equilibrium due to the increased intestinal permeability in CD may cause a flow of active molecules to the liver through the portal circulation [34]. Liver exposure to high levels of angiogenic factors could lead to the development of vascular lesions. However, in this study, data supporting this hypothesis are lacking, because blood and mucosal VEGF and other proangiogenic cytokines were not determined. Kitajima et al. [44] studied a transgenic rabbit model with increased liver expression of the human VEGF, whose upregulation has already been reported in association with hemangiomas. In that study, the overexpression of VEGF in the liver was determined to be sufficient for inducing hemangiomas. Moreover, transgenic rabbits developed hemolytic anemia, thrombocytopenia, and splenomegaly, all possible features of genetic syndromes such as Kasabach-Merrit syndrome [7]; thus, the above model could be useful in elucidating the mechanism underlying the development of HH and related complications.

Finally, another possible explanation could be a common genetic pathway, since familial or genetic patterns of inheritance have been hypothesized in both CD and HH. CD usually develops in genetically predisposed patients, with a strong association with HLA-DQ genes [13]. On the other hand, HHs have been described as part of defined clinical syndrome and, according to some authors, hemangiomas may be indicators of serious syndromes. However, no patient included in this study was affected by a genetic syndrome.

## 5. Conclusions

Overall, as from our data, HH prevalence seems to be significantly higher in CD patients than in the general population. Further studies are needed both to confirm this data and to explain the possible pathogenetic pathways, before suggesting that all patients with US findings consistent with HH should be screened for CD.

## Figures and Tables

**Table 1:** Demographic and clinical characteristics in celiac disease (CD) patients and controls.

	CD (#97)	Controls (#1352)	*P*
Age (yrs), median (range)	41 (17–84)	50.5 (16–94)	n.s.
Female, # (%)	79 (81)	771 (57)	<0.0001
BMI^§^ 25–30, # (%)	4 (4.1)	63 (4.7)	n.s.
Albumin (g/dL), median (range)	4.3 (3.8–5.1)	4.6 (4–5.1)	n.s.
Steatosis, # (%)	36 (37)	469 (34.6)	n.s.
Grades 1, 2, and 3^*^	29, 5, 2	236, 181, 52	

^§^BMI (body mass index) kg/m^2^.

^*^Grade 1: attenuation in the posterior segments of the liver; Grade 2: loss of echoes from the diaphragm; Grade 3: loss of echoes from the walls of the portal vein.

**Table 2:** Characteristics of US findings in celiac disease (CD) patients and in controls.

	CD (#97)	Controls (#1352)	*P*
HH, # (%)	14 (14.4)	69 (5.1)	0.0009
Prevalence of HH by gender			
Females, # (%)	13/79 (16.4)	46/771 (5.9)	0.002
Males, # (%)	1/18 (5.6)	23/581 (3.9)	n.s.
Number of lesions			
Single, # (%)	12 (85.7)	59 (85.5)	n.s.
Multiple, # (%)	2 (14.3)	10 (14.5)	n.s.
HH diameter (cm); median (range)	1.3 (0.3–6.8)	1.5 (0.7–4.6)	n.s.
Atypical features^*^, # (%)	1 (1)	11 (0.81)	n.s.

HH: hepatic hemangioma.

^*^Hypoechoic or isoechoic lesions, mixed echogenicity, lobulated mass, and blurred margins.
